# False positive malaria rapid diagnostic test in returning traveler with typhoid fever

**DOI:** 10.1186/1471-2334-14-377

**Published:** 2014-07-09

**Authors:** Bonnie Meatherall, Keith Preston, Dylan R Pillai

**Affiliations:** 1 Department of Medicine, University of Calgary, Calgary, AB, Canada; 2 Calgary Laboratory Services, Calgary, AB, Canada; 3 Department of Pathology and Laboratory Medicine, University of Calgary, Calgary, AB, Canada; 4 The University of Calgary, Diagnostic & Scientific Centre, Room 1 W-416, 9-3535 Research Road NW, Calgary, AB T2L 2 K8, Canada

**Keywords:** Malaria, False positive, Rapid diagnostic test, Typhoid

## Abstract

**Background:**

Rapid diagnostic tests play a pivotal role in the early diagnosis of malaria where microscopy or polymerase chain reaction are not immediately available.

**Case presentation:**

We report the case of a 39 year old traveler to Canada who presented with fever, headache, and abdominal pain after visiting friends and relatives in India. While in India, the individual was not ill and had no signs or symptoms of malaria. Laboratory testing upon his return to Canada identified a false positive malaria rapid diagnostic (BinaxNOW® malaria) result for *P. falciparum* with coincident *Salmonella Typhi* bacteraemia without rheumatoid or autoimmune factors. Rapid diagnostic test false positivity for malaria coincided with the presence or absence of *Salmonella Typhi* in the blood.

**Conclusions:**

Clinicians should be aware that *Salmonella Typhi* infection may result in a false positive malaria rapid diagnostic test. The mechanism of this cross-reactivity is not clear.

## Background

Diagnosis of malaria relies on both rapid diagnostic tests and thick and thin film microscopy for confirmation. RDTs have the advantage that no significant expertise is required and the test can be performed at the point of care. Delays in reading the peripheral smear can mean that clinicians rely on the RDT for initiation of therapy and early management.

## Case presentation

A 39 year old who travelled from Canada to India for two weeks developed acute onset of fever upon returning to Canada. He was born in India and lived there until the age of 27 at which point he emigrated to Canada. He returned to India on an emergent basis for a family member’s funeral. He did not take malaria prophylaxis nor did he take anti-malarial medication on this occasion but had on previous trips to India where he returned to on a yearly basis to visit friends and relatives (VFR). He was otherwise healthy and does not take any medications chronically. He denies any previous travel-related illnesses. He had not received typhoid vaccination prior to this trip or in the past.

**Figure 1:**
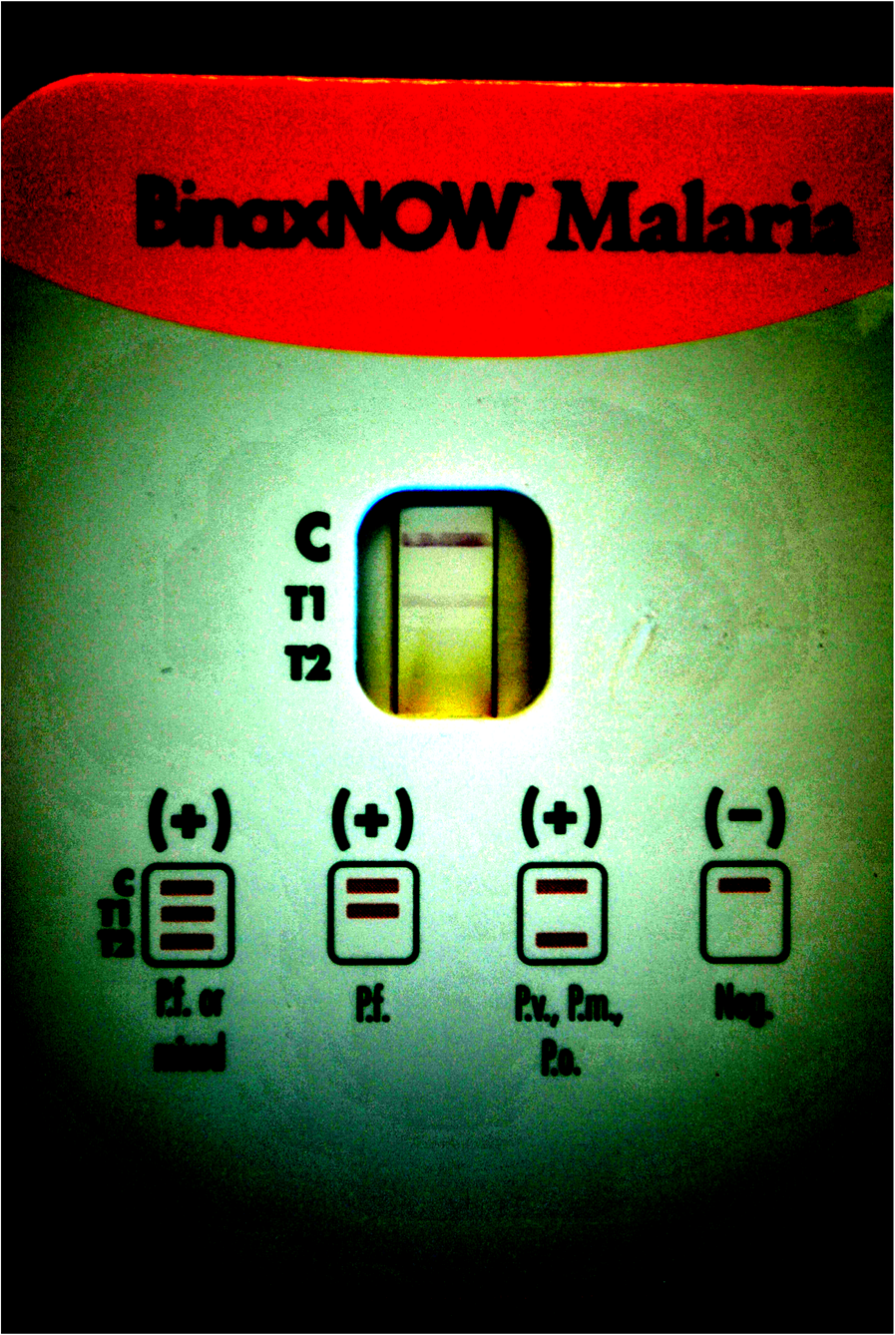
**Photograph of BinaxNOW® malaria RDT result from Day 37 showing a positive band at position T1 corresponding to *P. falciparum *infection.** The photograph is contrast enhanced to ensure the bands can be seen by the reader.

The patient developed fever, headache and abdominal pain 13 days after returning to Canada. He presented to the emergency department on day seven of illness (day 0 of laboratory testing). A rapid diagnostic test (RDT) for malaria was used during his initial diagnostic work-up for acute fever in the returning traveler, which was positive for *P. falciparum* (Figure 1). The patient received a three day course of oral atovaquone-proquanil (Malarone®) for presumptive infection with malaria starting day 7 of illness. However, subsequent thick and thin peripheral blood smears and PCR testing [1] were negative for malaria on the day 0 specimen.

Blood cultures were drawn on the day of presentation when Malarone® was initiated and were positive for *Salmonella Typhi* with intermediate susceptibility to ciprofloxacin (E-test), but susceptible to trimethoprim-sulfamethoxazole (TSX) and ampicillin (Vitek2, Biomerieux) based on Clinical Laboratory Standard Institutes testing guidelines [2]. He was referred to the outpatient adult infectious diseases clinic for treatment with three days intravenous Ceftriaxone followed by two weeks of oral TSX with complete resolution of his symptoms. Follow up blood cultures on day 4 and 20 were negative. Unfortunately, three weeks after completing therapy, he developed recurrence of fevers and his blood cultures were again positive for *S. Typhi* on day 37 without evidence for gallbladder disease based on abdominal ultrasound. He was then successfully treated for a relapse of typhoid fever with two weeks intravenous Ceftriaxone through a home parenteral therapy program with a peripherally inserted central catheter.

**Figure 2:**
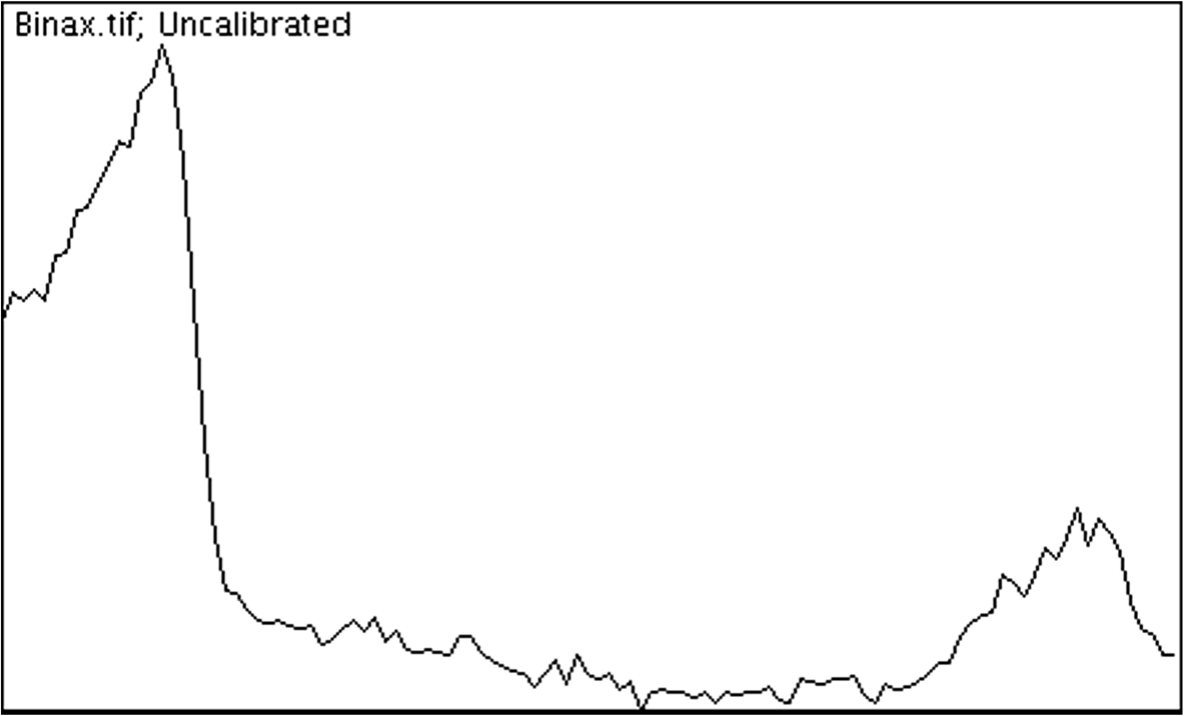
**Denistometry of the control band (leftmost peak) and T1 band (second peak) showing a signal present compared to the background for the RDT result.** The y-axis measures pixel intensity versus the distance on the RDT strip. Densitometric analysis was performed using NIH Image J software (http://rsb.info.nih.gov/ij/) [5].

His malaria RDT was negative on day 4 but reverted positive on two further occasions (day 20, day 37) within approximately six weeks of the initial positive RDT, all with negative thick and thin smears (Table 1). On two of the three occasions that false positive RDT results were obtained, *S. Typhi* was concomitantly observed in positive blood cultures. We explored the possibility that the patient was positive for rheumatoid factor (RF) which could lead to a false positive RDT per the product insert and previous literature. RF, as well as other auto-immune markers, was negative (Table 2). IgG and IgM to dengue virus were positive. The BinaxNOW ® malaria RDT is the only Food and Drug Administration (FDA)-approved rapid test for malaria in North America [3]. The test is an immunochromatographic membrane that uses monoclonal antibodies directed against *P. falciparum* histidine rich protein 2 (hrp 2, T1 band) and a pan-malarial (non-falciparum) antigen (aldolase, T2 band) to identify the presence other malaria species (Figure 1). In our patient, the test was positive at the T1 band suggestive of *P. falciparum* infection, however expert microscopy and PCR (more sensitive tests) failed to confirm this result. The band at the T1 position was confirmed by densitometry analysis (Figure 2). Moreover, concomitant *S. Typhi* was identified in blood cultures on two of three occasions when the RDT was positive during the course of the patient’s illness. This raises the possibility of a false positive rapid test caused by the presence of interfering substance related to typhoidal infection since the patient had no history of malaria while in India or prior antimalarial chemotherapy. We do note that standard strains of *Salmonella* (*Typhi* and non-*Typhi*) when inoculated directly on to the RDT do not result in a T1 band, suggesting that the interfering substance is in the blood itself rather than derived from the microorganism (data not shown). According to the product insert, RF and human anti-mouse antibody (HAMA) were the only interfering substances that caused a false positive [4]. We therefore tested our patient for a full panel of auto-immune antibodies and found none. The patient was also on no medication at the time of initial investigation for malaria that could interfere with the test. The product insert also confirms that seven bacteria, five protozoa, and sixteen viruses including four separate dengue virus subtypes did not interfere with the test. However, *Salmonella spp*, a common cause of non-malaria febrile illness (NFMI), in returning travelers was not reported.

**Table 1:** Results of BinaxNOW® malaria RDT, peripheral blood smear, PCR, and blood cultures

**Day after presentation**	**BinaxNOW®**	**Peripheral blood smear**	**Malaria PCR**	**Blood cultures**
	**Malaria (T1)**			
Day 0	POS^#^	NEG	NEG	*S. Typhi*
Day 4	NEG	NEG	NA	NEG
Day 20	POS^#^	NEG	NA	NEG
Day 37	POS^#^	NEG	NA	*S. Typhi*
Day 41	NA	NA	NA	NEG

POS- positive, NEG- negative, NA – test not performed; # T1 band only. Each positive RDT result is from a different lot number for the BinaxNOW® assay.

**Table 2:** Auto-immune profile of patient

**Test**	**Result**
Rheumatoid factor < 10 kU/L (normal range 0-20 kU/L)	Negative
Anti-nuclear antibody	1:80 (Negative)
Rh blood type	Positive
Anti-Jo-1	Negative
Anti-RNP	Negative
Anti Scl-70	Negative
Anti-Sm	Negative
Anti SS-A/Ro 60	Negative
Anti Ro 52	Negative
Anti SS-B/La	Negative
Anti-Ribo-P	Negative

Persistent hrp 2 antigen (up to 27% at day 28) after successful treatment of malaria is well documented both in endemic settings and in returning travelers [6,7]. Furthermore, false positives for hrp 2 have been documented for patients with RF and co-incident parasitic infections such as schistosomiasis and toxoplasmosis, as well as viral infections such as hepatitis C virus [8]. Generally, the RDT has been reported to work effectively (84.2% sensitive and 99.8% specific) in a U.S. teaching hospital when compared to microscopy [9]. In a study conducted in Tanzania with a related RDT (Paracheck®) which also relies on hrp 2, the adjusted odds ratio of obtaining a positive RDT with a negative peripheral blood smear examination was 3.82 (2.25-6.50, *N* = 169) in the presence of non-*Typhi Salmonella* bacteraemia, as opposed to 1.52 with any invasive bacterial disease [10]. These data suggest that co-infection with sub-microscopic *P. falciparum* occur with *Salmonella spp.* bacteraemia commonly in endemic areas. It is not clear whether some of these are false positive RDT results due to an interfering substance associated with *Salmonella spp.* bacteraemia. It has been reported that up to three quarters of *Salmonella* bacteraemia patients have been shown to be either recently or concurrently infected with malaria [11]. However, studies that rely on the hrp 2 antigen alone and not peripheral smears (thick and thin blood films) and PCR to determine recent malaria infection may over-estimate numbers due to false positives. That is to say, false positivity with malaria RDTs due to persistent hrp 2 antigenemia after recent malaria infection, interfering substances such as RF or another pathogen, or actual co-infection will over-estimate rates of malaria. Moreover, it is difficult to tease out these phenomena in malaria and typhoid co-endemic settings.

## Conclusions

In our patient, we cannot rule out earlier *P. falciparum* infection that cleared with partial immunity in this individual who was born in India. However, we do note that the RDT turned negative on day 4 when blood cultures transiently cleared on ceftriaxone. Clinicians who order blood cultures in addition to malaria testing in returning travelers to a non-endemic setting who present with fever, should be aware of possible false positive reactions in patients with Salmonellosis (typhoidal and non-typhoidal). It is not clear whether the Salmonella retains an antigen that mimics hrp2 or whether antibodies from the septic patient create false positive reactions through anti-idiotypic antibody reactions. Interestingly, the patient also had positive IgG and IgM to dengue virus during this episode, suggesting the individual was mounting a broad antibody response to several pathogens, possibly non-specific in nature as he had no classic dengue-like symptoms and PCR testing for dengue virus was negative. Of note, when the RDT is performed, whole blood (EDTA tube) is used containing the plasma where interfering antibodies reside. We conducted a retrospective analysis of the all RDT (n = 4514) performed on malaria suspected patients from 2008 - 2012 in our laboratory and found no other false positive T1 bands co-incident with *S. Typhi* bacteriemia. Therefore, the phenomenon observed with this patient appears to be a rare event. The possibility of a faulty BinaxNOW® lot was ruled out since each of the three positive results was from different lot numbers (057580, 061868, and 061318). This case reinforces the importance of expert microscopy and/or PCR in the confirmation of presumptive results with the RDT. It has been demonstrated previously that expert microscopy and PCR have superior analytical sensitivity to RDTs using latent class and receiver operating curve analysis [12]. A limitation of our study was that PCR was not performed on subsequent specimens from the same patient per our current testing algorithm in order to save costs. However, the likelihood of a positive PCR result on subsequent specimens is unlikely because the excellent sensitivity of our PCR assay [1]. We recommend that diagnosticians be aware that *Salmonella* bacteraemia may cause a false positive reaction in VFR and returning travelers who present with febrile illness of unknown etiology, and in surveillance studies conducted in endemic regions. False-positives can lead to unnecessary administration of potentially harmful drugs to the patient and misdiagnosis in surveillance of malaria if used as the sole diagnostic modality.

## Consent

Written informed consent was obtained from the patient for publication of this Case report. A copy of the written consent is available for review by the Editor of this journal.

## Competing interests

The authors declare that they have no competing interests.

## Authors’ contributions

BM assessed and managed the care of the patient. DP and KP were responsible for overseeing the diagnostic testing. Both BM and DP contributed to the writing of the manuscript. All authors read and approved the final manuscript.

## Pre-publication history

The pre-publication history for this paper can be accessed here:

http://www.biomedcentral.com/1471-2334/14/377/prepub
